# A model for genesis of transcription systems

**DOI:** 10.1080/21541264.2015.1128518

**Published:** 2016-01-06

**Authors:** Zachary F. Burton, Kristopher Opron, Guowei Wei, James H. Geiger

**Affiliations:** a Department of Biochemistry and Molecular Biology, Michigan State University, E. Lansing, MI, USA; b Department of Mathematics, Michigan State University, E. Lansing, MI, USA; c Department of Chemistry, Michigan State University, E. Lansing, MI, USA

**Keywords:** cradle-loop barrel metafold, double-Ψ−β-barrels, general transcription factors, LECA (the last eukaryotic common ancestor), LUCA (the last universal common cellular ancestor), replication, RIFT barrels, RNA polymerase, Rossmann folds, σ factors, TATA-binding protein (TBP), the carboxy terminal domain (CTD) of RNA polymerase II, TIM barrels, transcription, transcription factor B (TFB), α/β protein folds

## Abstract

Repeating sequences generated from RNA gene fusions/ligations dominate ancient life, indicating central importance of building structural complexity in evolving biological systems. A simple and coherent story of life on earth is told from tracking repeating motifs that generate α/β proteins, 2-double-Ψ−β-barrel (DPBB) type RNA polymerases (RNAPs), general transcription factors (GTFs), and promoters. A general rule that emerges is that biological complexity that arises through generation of repeats is often bounded by solubility and closure (i.e., to form a pseudo-dimer or a barrel). Because the first DNA genomes were replicated by DNA template-dependent RNA synthesis followed by RNA template-dependent DNA synthesis via reverse transcriptase, the first DNA replication origins were initially 2-DPBB type RNAP promoters. A simplifying model for evolution of promoters/replication origins via repetition of core promoter elements is proposed. The model can explain why Pribnow boxes in bacterial transcription (i.e., ^−12^TATAATG^−6^) so closely resemble TATA boxes (i.e., ^−31^TATAAAAG^−24^) in archaeal/eukaryotic transcription. The evolution of anchor DNA sequences in bacterial (i.e., ^−35^TTGACA^−30^) and archaeal (BRE_up_; BRE for TFB recognition element) promoters is potentially explained. The evolution of BRE_down_ elements of archaeal promoters is potentially explained.

After the advent of coding, ancient evolution of life on earth becomes a starkly simple story of replication errors or, perhaps more likely, RNA gene fusions/ligations resulting in repeating RNA sequences encoding repeating protein motifs.^1^ A trend toward increased biological complexity was largely driven by generation of repeating sequences, for which there appears to have been strong positive selection. The number of repeats often appears to be limited by solubility and structural closure, and also some dimeric repeats or true dimers were selected for nucleic acid binding (i.e., TBP and helix-turn-helix dimers). Because of relatively weak initial competition for enzyme specificity and functionality from ribozymes, many of the earliest successful protein folds were strongly selected for structure, solubility, and complexity. The ancient and ubiquitous α/β protein fold that supports most of fundamental metabolism and energy transduction appears to have been initiated by repetition of a β−α−β−α motif (Fig. 1). During emergence from the RNA-protein world (∼4.1 billion years ago) through LUCA (the last universal common cellular ancestor of bacteria, archaea and eukaryotes; ∼3.5 to 3.8 billion years ago), 2-double-Ψ−β-barrel (DPBB) type RNA polymerases (RNAPs) remained a major replicating polymerase.^2-6^ LUCA evolved to become one of the first cellular organisms with a unified DNA genome. Replication of the first DNA appears to have been initiated using 2-DPBB type RNAPs followed by DNA synthesis using reverse transcriptase.^7^ Because 2-DPBB type RNAPs dominated LUCA replication and transcription, divergence of bacteria and archaea was driven by coevolution of 2-DPBB type RNAPs, RNAP general transcription factors (GTFs) and RNAP promoters. By contrast, DNA polymerases (DNAPs) and distinct promoters and replication origins were not yet dominant.^7^ In support of this ancient replication mechanism, non-homologous DNAPs that exist today in bacteria and archaea appear to have arisen separately after divergence of bacteria and archaea.

**Figure 1.**
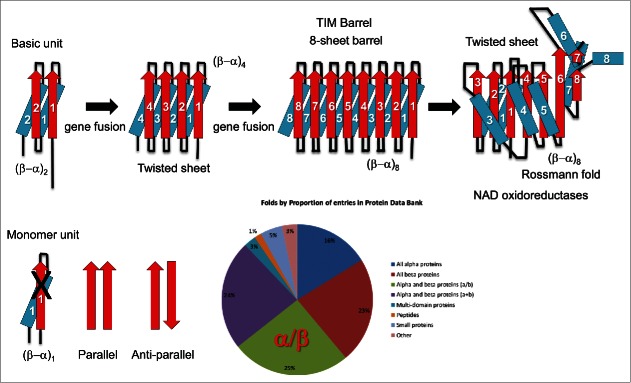
α/β folds are simple (β−α)_n_ repeat proteins. The pie chart indicates that ∼25% of all structures in the RCSB protein data bank are α/β fold proteins. A model is shown for evolution of TIM barrels (β−α)_8_ and Rossmann folds (β−α)_8_.

Eukaryotes are generally more complex than bacteria and archaea, and the tortured path to eukaryotic evolution explains increased genomic, functional and organismal complexity. At LECA (the last eukaryotic common ancestor; ∼1.6 to 2.2 billion years ago), eukaryotes resulted from endosymbiosis and genetic fusion of a Lokiarchaeota phylum archaea^8,9^ and an α-proteobacterium.^10,11^ Although many modern archaea have lost the capacity to engulf a bacterial endosymbiont, Lokiarchaeota has the ESCRT I, II and III (endosomal sorting complexes required for transport) endocytosis/phagocytosis systems and also actin and tubulin, which are also required for endocytosis (Fig. S1).

In eukaryotes, genetic duplications generated RNAPs I, II and III and the carboxy terminal domain (CTD) repeat on RNAP II, which facilitated nuanced RNAP II regulation required to support complexity and multicellularity.^4^ The story of the inception of biological complexity, therefore, includes recurrent cases in which repeating sequences were generated.^1^ Most surprisingly, however, after up to ∼3.5 to 4 billion years, initial repeats can remain recognizable, sometimes in sequence but more often in secondary structure. Examples include α/β proteins, the RIFT barrel, DPBBs, bacterial σ transcriptional initiation factors, TFB (transcription factor B), TBP (TATA-binding protein) and the RNAP II CTD. Core promoter elements are posited also to be generated via repetition of motifs, potentially explaining the similarity between archaeal/eukaryotic TATA boxes (i.e., ^−31^TATAAAAG^−24^) and bacterial Pribnow boxes (i.e., ^−12^TATAATG^−6^) (see below).

## α/β folds: generating complexity via RNA gene fusions/ligations

Remarkably, glycolysis, the citric acid cycle and the glyoxylate cycle are catalyzed by ancient α/β fold proteins (Fig. 1, Figs. S2-S8). In addition to core metabolism and redox potential, ATPases, GTPases and kinases are supported by α/β folds. So, much or all of the most ancient metabolism and also redox and chemical energy transduction are supported by α/β fold proteins that date from the RNA-protein world. The α/β proteins can be described as (β−α)_n_ repeat proteins, generated from β−α−β−α repeats.^12,13^ Because to support an extended chain structure a β-sheet requires hydrogen bonding to a second β-sheet, the basic unit for repeat generation appears to be β−α−β−α rather than a monomeric β−α unit. A β-sheet interacts with a neighboring β-sheet in either a parallel or antiparallel orientation, and, without a partner, a β-sheet cannot hydrogen bond to maintain its characteristic extended β-sheet conformation. Most ancient proteins with many parallel β-sheets are α/β fold proteins. As shown in Figure 1, the α/β fold comprises about 25% of proteins represented in the RCSB protein data bank. A model is shown for fusion of (β−α)_n_ repeats to generate TIM barrels (β−α)_8_ and Rossmann folds (β−α)_8_. We conclude that ubiquitous α/β fold proteins are generated from simple (β−α)_n_ repeats.

Glycolytic enzymes are of the TIM (triose phosphate isomerase) barrel fold (β−α)_8_ (Fig. S2).^14-21^ This ancient and ubiquitous fold was generated by duplication (probably via RNA gene fusion or ligation) of a (β−α)_4_ unit, which was itself generated by earlier duplication of a (β−α)_2_ unit. Formation of the 8-parallel β-sheet TIM barrel gives closure to the structure. Further polymerization beyond 8 sheets breaks the barrel and can create a larger, more flexible horseshoe structure. Compared to TIM barrels, Rossmann folds are generated from (β−α)_8_ repeats that are rearranged into a twisted sheet (Figs. 1 and S3).^15,19,22-24^ Starting from a TIM barrel, in order to generate a Rossmann fold required rearrangement of β4 to pair with β1 rather than with β3. Such rearrangement is possible because α3 and its surrounding loops can span the distance required for repositioning β4. A second rearrangement pairs both β7 and β8 with β6, as indicated in Figures 1 and S3. We posit that Rossmann folds (β−α)_8_ may have resulted from rearrangement of a TIM barrel (β−α)_8_. In a TIM barrel, β1−β8 curvature supports closure of the barrel. Because of necessary rearrangements, the Rossmann fold linear, twisted sheet is supported by the opposite curvature of β1−β3 and β4−β7/β8. Similarly, TOPRIM domains ∼(β−α)_4-5_ (Fig. S4) appear to be generated from a larger repeat such as a Rossmann-like fold. Many ATPases, GTPases and kinases are Rossmann-like folds that emerged in the RNA-protein world (Fig. S5-S6). Swi-Snf ATPases include 2 (β−α)_6_ domains (Figs. S7-S8). Some of these folds require rearrangements of β-sheets. Ubiquitous α/β fold proteins that account for essentially all ancient metabolism and energy transduction, therefore, were generated on a scaffold formed by simple repetition of a β−α−β−α unit.

Importantly, α/β protein folds provide both structure and solubility. The β−α−β−α unit and its larger repeats create structure through interaction of parallel β-sheets, and these folds are soluble, because β-sheets, which by themselves might form amyloid-like interactions, are each paired with an α-helix. The solubility of the β-sheet fold, therefore, appears to have been promoted by the associated helices. Early and enduring success of the α/β fold, therefore, is explained by structure, sufficient functionality and solubility. Remarkably, so far as we are aware, the active site in α/β fold proteins is always located to the C-terminal side of the β-sheets that dominate the fold. It appears that this relation may have been established ∼4 billion years ago on a ∼4.6 billion year old earth and maintained via powerful co-evolutionary forces. So far as we are aware, in a TIM barrel, a Rossmann fold or a Rossmann-like protein, no active site or allosteric site locates to the N-terminal end of the β-sheets.

## A simple model for evolution of RNAPs, general transcription factors and promoters: Complexity generated via repeated sequences

### Two-DPBB type RNAPs

**Figure 2.**
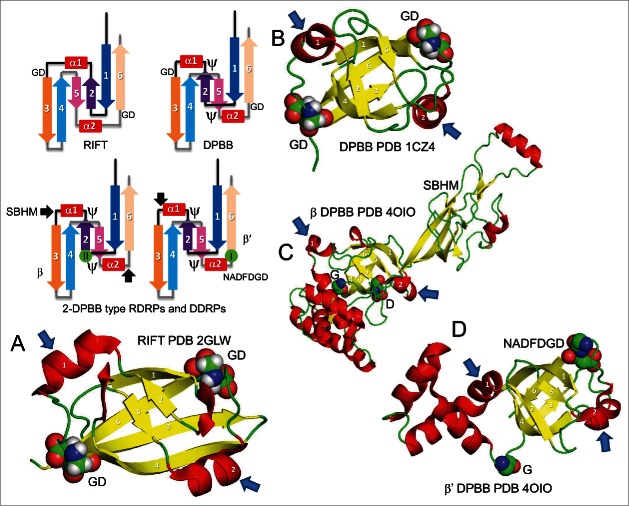
Cradle-loop barrels: RIFT barrels and DPBBs. A) PHS018 RIFT barrel (PDB 2GLW).^26^ B) VatN-N DPBB (PDB 1CZ4) (a AAA+ ATPase).^54^ C) RNAP β’ DPBB (PDB 4OIO).^55^ D) RNAP β DPBB (PDB 4OIO). Small blue arrows indicate α1 and α2. Small black arrows in the schematics indicate insertions in RNAP DPBBs. Conserved GD motifs and possible GD relics in RNAP DPBBs are indicated in sphere representation. A signature motif of RNAPs, NADFDGD that binds Mg-I (Mg-A), ends in a conserved GD box. Molecular graphics images were made using Pymol (https://www.pymol.org/).

Multi-subunit RNAPs are of the 2-DPBB type.^2,4-6^ DPBBs are 6-β-sheet barrels of the ancient cradle-loop barrel metafold.^25^ In Figure 2, schematic diagrams are shown of the DPBB and its parent, the RIFT barrel (RIFT for its occurrence in riboflavin synthases, F1 ATPase and translation factors).^25-27^ RIFT barrels were initially generated from dimerization of a β1−β2−α1−β3 motif, which subsequently became ligated to form a β1−β2−α1−β3−β1′−β2′−α1′−β3′ barrel. Unlike the RIFT barrel, the DPBB has a complex looped and pseudo-knotted fold. Monomeric RIFT barrels formed from dimeric RIFT barrels through duplication probably via ligation of 2 identical RNAs. Dimeric RIFT barrels gave rise to 8-β-sheet swapped hairpin barrels (not shown). The coloring of the schematic in Figure 2 was chosen to emphasize the duplication or gene fusion. In molecular graphic images (Figs. 2A-D), features of the specialized RNAP DPBBs are emphasized. In RIFT barrels and DPBBs, the conserved GD (glycine-aspartic acid) box is found after α1 and α2 and just before β3 and β6. The signature motif of multi-subunit RNAPs, NADFDGD that binds the catalytic Mg-I (Mg-A) through 3 aspartic acids, appears to end in a GD box.^25^ In RIFT barrels and DPBBs, β2 and β5 lie in a “cradle” formed by β1, β6, β3 and β4. The β1-β2 and β4-β5 loops form the “cradle-loops” of the cradle-loop barrel fold.

**Figure 3.**
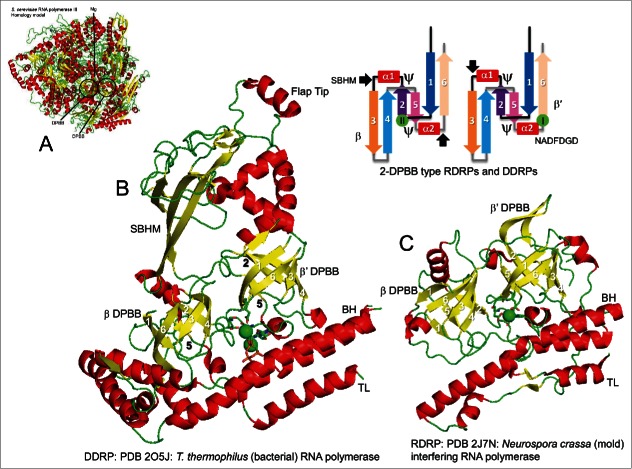
2-DPBB type RNAPs. A) *S. cerevisiae* (yeast) RNAP III (a homology model). The 2-DPBBs border the active site. B) *Thermus thermophilus* RNAP catalytic core including 2-DPBBs, the SBHM, the bridge helix (BH) and trigger loop (TL) (PDB 2O5J) (a DNA template-dependent RNAP).^31^ C) *N. crassa* (mold) interfering RNAP catalytic core including 2-DPBBs, BH and TL (PDB 2J7N) (a RNA template-dependent RNAP).^34^

In RNAPs, 2-DPBBs border the 2-Mg active site, and loops from the barrels bind active site Mg-I and Mg-II (Mg-A and Mg-B) (Fig. 3). Opposite from the DPBBs, the bridge helix and the mobile trigger loop also enclose active site Mg-I and Mg-II. Because multi-subunit RNAPs distribute to all cellular life, 2-DPBB type RNAPs must have been present at LUCA (∼3.5 to 3.8 billion years ago).^2,4^ Because both DNA template-dependent and RNA template-dependent RNAPs of the 2-DPBB type exist, 2-DPBB type RNAPs appear to be rooted in the RNA-protein world (up to ∼4.1 billion years ago) prior to LUCA (>3.5 billion years ago). Interestingly, in transcription and replication of Hepatitis δ virus, which has a RNA genome, human RNAP II, which is normally a DNA template-dependent RNAP, can function as a RNA template-dependent RNAP.^28,29^ Unique to DNA template-dependent RNAPs and inserted between β2 and β3 of the β-subunit type DPBB is a sandwich barrel hybrid motif (SBHM) that permits utilization of a DNA template for initiation and elongation.^2,4^ To illustrate this point, in bacterial RNAP, the SBHM is also termed the “flap” domain. The “flap tip” helix interacts with the bacterial σ factor (HTH_4_) to support transcription initiation^30^ and, during elongation, interacts with the exiting RNA.^31,32^ To overcome transcription stalls, the flap tip helix also interacts with the Swi-Snf type ATPase HepA/RapA to promote RNAP backtracking.^33^ 2-DPBB type RNA template-dependent RNAPs include a bridge helix and a trigger loop, but lack an inserted SBHM, which is required for utilization of a DNA template but not an RNA template.^34^

### General transcription factors (GTFs)

#### TBP and σ/TFB

**Figure 4.**
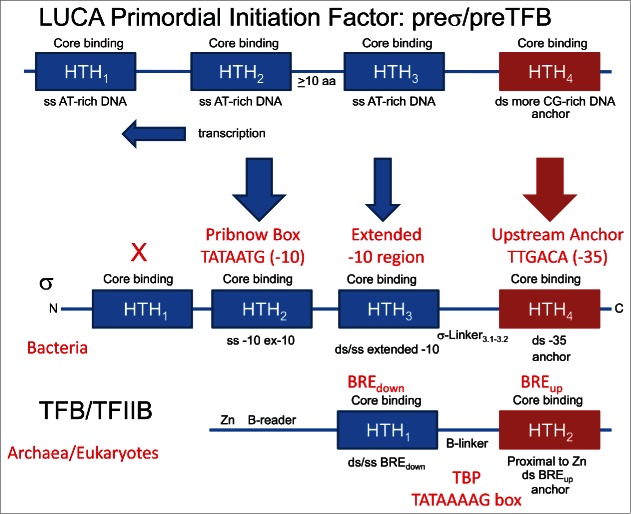
A model for evolution of bacterial σ factors and archaeal TFB from a 4-HTH primordial initiation factor at LUCA. Classic σ homology regions overlap with HTH_1-4_.^35^

Here we describe evolution of TBP (TATA-binding protein) and a primordial transcription initiation factor that gave rise to σ factors in bacteria and to TFB (Transcription Factor B) in archaea, driving divergence of bacteria and archaea.^35^ TBP includes 2 TBP-fold repeats. TFB, with 2-HTH (helix-turn-helix) repeats, appears to be derived from a 4-HTH primordial initiation factor. Both TBP and the 4-HTH primordial initiation factor are posited to have existed at LUCA.^35^ As described above, at LUCA, GTFs can also be considered replication origin binding factors, because replication on the first DNA templates initiated via transcription followed by reverse transcription.^7^ TBP was generated via duplication of a TBP fold, and, consistent with its near 2-fold symmetry, TBP lands within the DNA minor groove at the TATAAAAG box.^36^ As we have described elsewhere, the primordial initiation factor that gave rise to σ/TFB is posited to be a regular repeat of 4-HTH domains (Fig. 4). In sequence, the primordial initiation factor is most similar to the 2-HTH domains (historically termed “cyclin-like” repeats) of archaeal TFB. Because of cooperation and compensation from TBP and TFE, and because of addition to TFB of a N-terminal Zn-ribbon extension, 2 of 4-HTH units are thought to have been lost from TFB in evolution. Bacterial σ factors are derived from a repeat of 4-HTH domains, but, because of coevolution with RNAP and promoters, the 4-HTH repeat structure in σ, although recognizable, is degenerate in sequence.^35^

**Figure 5.**
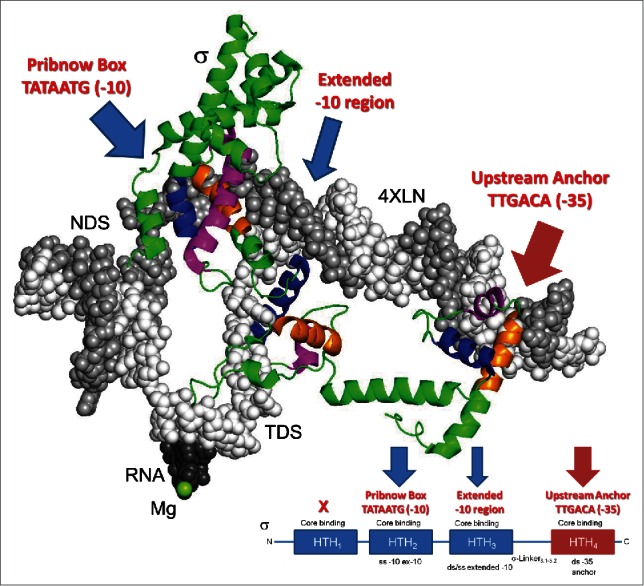
Bacterial σ factor interactions with promoter DNA in initiating complexes with an open transcription bubble (PDB 4XLN).^37^ RNAP was removed from the image in order to visualize σ (green except at HTH motifs) interactions to promoter DNA. HTH units are colored blue (H1), magenta (H2) and orange (H3; N-terminal end only). Only σ HTH_4_, HTH_3_ and HTH_2_ were colored. In this view, HTH_1_ is obscured by HTH_2_. To locate the RNAP active site, RNA and Mg-I are shown.

The evolutionary model for σ combined with recent x-ray structures of initiating RNAP describes σ functions in initiation. In Figure 5, a RNAP-promoter complex (PDB 4XLN) is shown with RNAP core subunits removed from the image to visualize the intact transcription bubble and DNA interacting with σ.^37^ Because HTH_1_ appears vestigial, HTH_1_ is not shown. HTH_4_ binds the “anchor” DNA -35 region of the bacterial promoter (^−35^TTGACA^−30^). This contact anchors RNAP at the promoter upstream and specifies the direction of bubble opening and downstream transcription. HTH_4_ is a typical HTH unit with a 8-10 amino acid H2 that makes canonical contact, via the N-terminal end of its “recognition helix” H3, to the major groove of the DNA. HTH_3_ is also a typical HTH that binds upstream of the initiation site where the bubble opens. HTH_3_ has a typical 8-10 amino acid H2. In the structure with an open bubble, the N-terminal end of HTH_3_ H3 does not bind in the DNA major groove, as would be expected during an initial encounter with double-stranded (ds) DNA, but no structure of HTH_3_ on dsDNA is currently available. Perhaps, as the bubble opens, HTH_3_ twists from an initial typical HTH-major groove contact. HTH_2_ is a highly specialized HTH-derived fold that opens the -10 region of the bacterial promoter (^−12^TATAATG^−6^). HTH_2_ has an elongated H2 (20 amino acids) and bulky hydrophobic residues on H3 (i.e., KFSTYATWWIR) judged inconsistent with binding to dsDNA.^38-40^ HTH_2_ aggressively attacks dsDNA, flips out ^−11^A of the non-template DNA strand (NDS) and then flips out ^−7^T on the NDS to help unzip DNA to +1 for initiation on the template DNA strand (TDS).^41^ Degeneracy of the 4-HTH units of σ relative to the proposed regular 4-HTH repeat LUCA primordial initiation factor, from which σ was derived, is explained by powerful coevolution of RNAP, σ HTH repeats and promoter DNA. To describe the evolutionary pressures on σ, each HTH repeat is selected for: 1) specific promoter recognition; 2) RNAP binding; 3) autoinhibition of promoter binding off of RNAP; 4) solubility off of RNAP; and 5) release from core RNAP during elongation. In σ factors, the 2 most important of the 4-HTH repeats are generally HTH_4_ that binds the anchor DNA (-35) and the highly specialized HTH_2_ that opens the -10 region.^39^ By contrast to σ, the 2 remaining archaeal TFB HTH repeats function cooperatively with TBP and TFE and more independently of RNAP, and, consistent with somewhat relaxed evolutionary pressures, TFB has maintained a very recognizable repeat structure (the 2 “cyclin-like” repeats) that through evolution have become partly obscured in σ. Although bacteria no longer utilize TBP, bacterial RNase HIII includes a TBP fold, indicating that a bacterial ancestor (i.e. LUCA) may have included TBP, as proposed here (Figure S9).^36,42^

### TFE

In addition to TBP and TFB, archaea also utilize the GTF TFEα/β.^43^ TFEα includes a winged HTH (WHTH) motif and a Zn ribbon. The WHTH motif in TFEα is most similar to the ArsR family of WHTH transcription factors dispersed to bacteria and archaea. The WHTH motif in TFEβ is most similar to the MarR family of WHTH transcription factors dispersed to bacteria and archaea. It is clear that the WHTH motif is ancient and, because it is distributed to both bacteria and archaea, the WHTH motif may have been present at LUCA. It is unclear when TFEα/β arose in archaea (i.e., before or after divergence of bacteria and archaea). Because of the difficulty in opening B-form DNA, it is also possible that a helicase may have aided promoter and replication origin opening at LUCA, analogous (not homologous) to the eukaryotic TFIIH helicases.

According to these simple and simplifying models, LUCA transcription and replication, on the first DNA templates, were supported by a mechanism that is very recognizable today. We posit that TBP bound multiple TATAAAAG boxes. A primordial initiation factor with 4-HTH repeats (“cyclin-like” repeats) bound to surrounding BREs (TFB-recognition elements). TFE may have been present, or TFE may have evolved separately in archaea after divergence. Also, a helicase may have facilitated promoter/replication origin opening. This model is simplifying, because it roots the tree of life at LUCA and describes the radiation of bacteria and archaea. Bacteria and archaea are posited to have diverged because they evolved to interpret, transcribe and replicate their genomes using distinct RNAP-GTF-promoter combinations. In bacteria, GTFs, RNAP and promoters became much more tightly coupled and co-dependent than in archaea, and this difference is seen comparing the degenerate HTH units of σ factors in bacteria and the more recognizable cyclin-like HTH repeats of TFB in archaea.

## Model for a LUCA promoter: Proposed TATAAAAG and BRE repeats, followed by simplification via coevolution in bacteria and archaea

**Figure 6.**
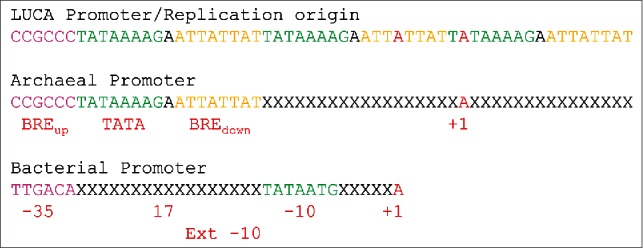
A model for a LUCA promoter sequence generated as an AT-rich repeat of TATAAAAG boxes and BREs. The repeat sequence simplifies to an archaeal and a bacterial promoter.

Repeating sequences generated α/β folds, RIFT barrels, DPBBs, TBP (2 TBP-fold repeats), σ (4-HTH repeats) and TFB (2-HTH repeats). Similarly, promoters at LUCA are posited to have evolved via repetition of an AT-rich sequence. As a possible example, a hypothetical LUCA promoter is posited to be generated by alternating repeats of a TATAAAAG box and an AT-rich BRE_down_ (TFB-recognition element downstream of TATA) (Fig. 6). Three repeats are shown with an upstream anchor sequence (a GC-rich BRE_up_). In the LUCA promoter/replication origin, there may have been many more than 3 repeats, but 3 repeats is sufficient to generate a model. From the proposed LUCA promoter, an archaeal promoter with a BRE_up_, a TATAAAAG box and a BRE_down_ can be generated via simplification, and simplification is also posited for archaeal TFB, which is posited to have lost 2-HTH repeats from a 4-HTH primordial initiation factor. Similarly, a bacterial promoter can be derived, with a ^−35^TTGACA^−30^ -35 anchor region, an extended -10 region, and a Pribnow box (^−12^TATAATG^−6^) -10 region. In this model, the Pribnow box of the bacterial promoter is derived from a downstream TATAAAAG box of the LUCA promoter, explaining why Pribnow boxes, which are bound by a specialized HTH-derived domain (σ HTH_2_), resemble TATAAAAG boxes, which are bound by TBP (not present in bacteria) binding in the DNA minor groove. Other features of promoters can be generated by coevolution of interacting factors and promoters. For instance, alternate σ factors with different promoter recognition can be generated via co-evolutionary forces. As we have previously shown, some alternate σ factors are most similar to archaeal TFB in sequence, particularly in σ HTH_4_ T1-H2-T2 (i.e., RRT-QREIAKAL-GIS) and TFB HTH_2_ T1-H2-T2 (i.e., RRT-QREVAEVA-GVT).^35^

### Model for LUCA GTFs on promoter DNA

**Figure 7.**
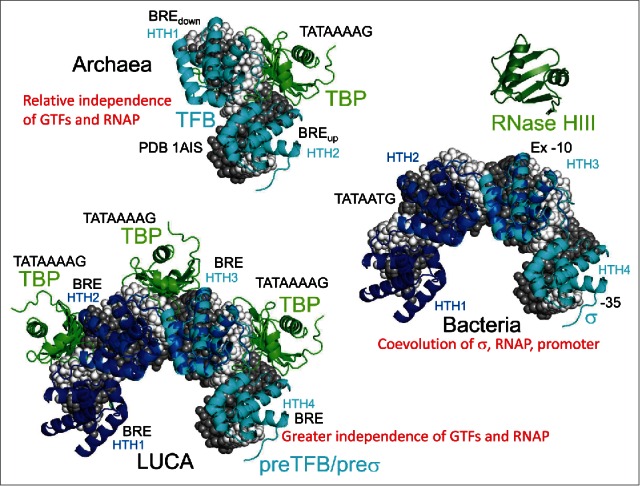
A model for primordial GTFs on a LUCA promoter and for radiation to archaea and bacteria. The model was constructed by superimposing 3-PDB 1AIS structures (archaeal TBP-TFB-promoter DNA).^44^ The 4-HTH primordial initiation factor was generated by sequential alignment of 3 2-HTH repeats of TFB HTH_1_-(HTH_2_/HTH_1_)-(HTH_2_/HTH_1_)-HTH_2_. Bacterial systems are posited to have lost TBP and to have made the σ factor more strongly coevolved with promoter DNA and RNAP than in archaea or at LUCA.

From X-ray structures, a model for GTFs on a LUCA promoter/replication origin can be constructed (Fig. 7). PDB 1AIS shows archaeal TBP and TFB HTH_1_-HTH_2_ on promoter DNA.^44^ The LUCA GTF-promoter model is obtained by superimposing 3-PDB 1AIS structures with TFB HTH_1_-(HTH_2_/HTH_1_)-(HTH_2_/HTH_1_)-HTH_2_. Because in the model 4 of 6-HTH units are superimposed (HTH_2_/HTH_1_), 6-HTH units reduce to 4-HTH units, as are found in bacterial σ factors. What results is a promoter repeat of 3-TATAAAAG boxes and 4-BREs. Three TBP molecules bind the 3-TATAAAAG boxes and the 4-HTH repeats each bind a BRE. Because the archaeal TBP-TFB-promoter 1AIS structure was used to generate the LUCA TBP-primordial initiation factor-promoter structure, archaeal transcription is obtained from the LUCA model by simplification. To suppress unwanted transcription starts in archaeal promoters, selection against multiple TATA boxes is expected, leading to degeneration of the initial repeat structure (Fig. 6). Bacteria do not utilize TBP, although bacteria encode RNase HIII, a TBP structural homolog (Fig. S9).^36,42^ As described above, bacterial σ factors are derived from a 4-HTH repeat primordial initiation factor. In archaeal transcription, GTFs are more independent of RNAP in the initiation mechanism and are not as powerfully coevolved as they are in bacteria, in which RNAP, the promoter and the σ factor are more strongly interacting and codependent. The degeneracy and specialization of the σ 4-HTH repeats, therefore, are explained by co-evolutionary pressures. In the LUCA model, TBP and the 4-HTH primordial initiation factor function as agents to facilitate DNA opening and appear more independent of RNAP than archaeal and bacterial GTFs. Because, without a mechanism to open DNA, DNA genomes cannot evolve from RNA genomes, TBP and the hypothesized 4-HTH primordial initiation factor are posited to be the most central components of this ancient mechanism, and TBP and the 4-HTH primordial initiation factor are posited to have been present at LUCA.^35^

To test the feasibility of this model, one might combine a LUCA promoter, LUCA GTFs and a 2-DPBB type RNAP on negatively supercoiled DNA and search for evidence of promoter opening and/or accurate initiation. A very similar experiment was successfully done decades ago using TBP, TFIIB and RNAP II on a supercoiled DNA template.^45^

### LECA: the RNAP II CTD repeat

LECA is the last eukaryotic common ancestor (∼1.6 to 2.2 billion years ago), and a surprisingly simple and compelling model for genesis of eukaryotes is available from extensive phylogenomic studies.^9-11,46-49^ A story of LECA, moreover, can be related by focusing on multi-subunit 2-DPBB type RNAPs and their associated factors (Figure S10). A current model is that eukaryotes arose from endosymbiosis between a eukaryote-like Lokiarchaeota phylum archaea^8,50^ and a resident population of α-proteobacteria. The mitochondria and mitochondrial DNA are relics of the α-proteobacteria. Many bacterial genes were transferred to what was initially the archaeal genome. Remarkably, a massive attack was launched against the archaeal genome by a α-proteobacterial group II self-splicing intron mobile genetic element.^10,47,51,52^ Eukaryotic splicing and widely dispersed introns developed from jumping and insertions of group II intron elements. Eukaryotic genome complexity, therefore, results (in part) from group II intron invasion, Lokiarchaeota DNA and α-proteobacterial DNA. Many other bacterial genomes are represented in eukaryotic DNA, presumably acquired from many horizontal gene transfers (i.e., from virus-mediated horizontal gene transfer, plasmid-mediated horizontal gene transfer, endosymbiosis and natural horizontal gene transfer). Of course, there are additional contributions to eukaryote genome complexity such as genome duplications, transposons and insertion elements. The nucleus arose as a defense against translation of intron sequences that invaded genes. The splicing apparatus is posited to have arose to restrain and regulate self-splicing of group II introns. Many other eukaryote-specific genes and functions evolved as a result of novel pressures from the initial genome fusion, intron invasion, new cell architectures and resulting chaos.

More complex eukaryotic genomes allowed for duplication of genes and diversification of functions (Figure S10). Two-DPBB type RNAPs diversified into RNAP I, II and III, and RNAP II acquired the carboxy terminal domain (CTD), a heptapeptide (7aa; consensus ^1^YSPTSPS^7^) repeat, on the largest (β’ type) subunit.^4,53^ RNAPs I, II and III and the CTD on RNAP II appear to be rooted in LECA. The CTD is thought to have initially evolved to couple splicing of introns to transcription, making the CTD YSPTSPS repeat another evolutionary defense against group II intron invasion.^53^ Subsequently, the CTD became a much more general scaffold for evolutionary innovation linked to RNAP II transcription. Ultimately, an extensive CTD interactome coupled transcription to many related processes. The CTD interactome interfaces with the chromatin interactome, linking transcription with epigenetics, and complex eukaryotic signaling is also coupled to the CTD and chromatin interactomes. Because of regulation by cyclins and cyclin-dependent kinases, the RNAP II transcription cycle, which is regulated by the CTD interactome, resembles a eukaryotic cell cycle, indicating that the RNAP II transcription cycle and the eukaryotic cell cycle were coevolved.^4^ Consistent with the complexity of the CTD and its interactome, regulation of RNAP II promoter-proximal pausing by the CTD and the CTD interactome appears to be a primary marker of animal body plan complexity (i.e., the pre-Cambrian and Cambrian Explosion). The simple idea is that ever more nuanced RNAP II regulation, centered in eukaryotes on the CTD interactome, licenses higher order organismal complexity. Consistent with this idea, the number of repeats found in the CTD in different eukaryotes tends to correlate with overall organismal complexity.^53^ According to this view, the CTD repeat initially evolved to cope with group II intron invasion and then became a scaffold for evolutionary innovation that was essential to support, and helped to drive, increasing eukaryote complexity. Landmark innovations in animal complexity, therefore, appear to track with innovations in RNAP II regulation via the CTD repeat interactome.

### Genesis of life on earth

Remarkably, the story of genesis of life on earth is told in stunning detail by tracking α/β fold proteins, 2-DPBB type RNAPs, RNAP GTFs, RNAP promoters, the CTD on RNAP II and the extensive CTD interactome. According to this view, repeating protein motifs (bounded by solubility) form a surprisingly dominant component of the fabric of life, as if, in early evolution, structural complexity may have been positively selected, even before active sites gained refined function and specificity. We suggest that some initial repeats must have had sufficient function to compete successfully with or to collaborate with ribozymes.

α/β proteins ((β−α)_n_ repeat proteins) have built-in structure through parallel interacting β-sheets and solubility through pairing of each β-sheet with its α-helix. As such, α/β proteins appear to have won a primordial race to structure, solubility and function. Remarkably, the β−α−β−α pattern remains discernable after almost ∼4.1 billion years of evolution on a ∼4.6 billion year old earth (Figs. S2-S8). This indicates, perhaps contrary to intuition, that evolution can be very conservative of core protein repeat motifs over a span of ∼4 billion years.

In unexpected ways, evolution describes transcription, and transcription describes evolution. Life originated from a RNA-protein world that included 2-DPBB type RNA-template dependent RNAPs, and, therefore, RNAPs are central to the story of life on earth from: 1) the RNA-protein world to LUCA; 2) from LUCA diverging to bacteria and archaea; and 3) from endosymbiotic fusion of a Lokiarchaeota and an α-proteobacterium at LECA. Thus, a stunningly simple working model for genesis of life on earth is available from evolution of 2-DPBB type RNAPs, RNAP GTFs, RNAP promoters, RNAPs I, II and III, and the RNAP II CTD (Figure S10). The model describes the major branch points in evolution and provides surprising insights into eukaryote and animal complexity. The model provides a deeply conceptual understanding of life on earth.

The complexity of protein structures and, therefore, of living systems can be bounded by the linked issues of: 1) solubility; and 2) closure. The repeating protein sequence folds that win tend to be soluble and to gain closure by forming pseudo-symmetric forms: i.e. dimeric repeats and tetrameric repeats. Examples of dimeric repeats: 1) double-Ψ−β-barrels; 2) 2-double-Ψ−β-barrel type RNAPs; 3) TBP (TATA-binding protein); 4) RIFT barrels; 5) TFB (Transcription Factor B); 6) TIM barrels (β−α−β−α−β−α−β−α dimer). Examples of tetrameric repeats: 1) the primordial initiation factor (4-HTH); 2) σ factor (4-HTH); 3) TIM barrels (β−α−β−α tetramer). Closure is attained by formation of barrels and/or pseudo symmetry. What this means is that there are limits to the complexity evolution will achieve, and limits are often approached by simple bounds of solubility and closure. The CTD (52 repeats in humans) is not strongly bounded by these rules because it is largely unstructured, attached to a soluble scaffold (RNAP II) and heavily modified. The length of the CTD is bounded by its functionality.^53^

## Supplementary Material

KTRN_A_1128518_Supplemental_Figures.pdf

## Abbreviations

CTD: carboxy terminal domain
DNAP: DNA polymerase
DPBB: double-Ψ-β-barrel
GTF: general transcription factor
LUCA: last universal common cellular ancestor of bacteria, archaea and eukaryotes
LECA: last eukaryotic common ancestor
RIFT: occurrence in riboflavin synthases, F1 ATPase and translation factors
TBP: RNA polymerase (RNAP); TATA-binding protein
TFB: Transcription Factor B
BRE: TFB-recognition element
TIM: triose phosphate isomerase
