# Supplementary material for: A model for genesis of transcription systems
Source: Transcription. 2016 Jan 6;7(1):1–13. doi: 10.1080/21541264.2015.1128518 (PMC4802759; doi:10.1080/21541264.2015.1128518)
Supplement: KTRN_A_1128518_Supplemental_Figures.pdf [file ktrn-07-01-1128518-s001.pdf]

A model for genesis of transcription systems

Zachary F. Burton[1,*], Kristopher Opron[2], Guowei Wei[2], James H. Geiger[3]

1. Department of Biochemistry and Molecular Biology, Michigan State University, E. Lansing, MI 48824-1319, burton@cns.msu.edu, 517-353-0859
2. Department of Mathematics, Michigan State University, E. Lansing, MI
3. Department of Chemistry, Michigan State University, E. Lansing, MI
   *Corresponding author

Supplementary Figures:

Figure S1. Eukaryotes are proposed to have been generated as an endosymbiotic fusion of a Lokiarchaeota archaea capable of endocytosis[1,2] and a α-proteobacterium.[3,4] Many modern archaea have lost the capacity for endocytosis. Red arrows indicate competition between eukaryotes and Lokiarchaeota, mostly won by eukaryotes. Most modern archaea may have lost the capacity for endocytosis, perhaps because of competition with eukaryotes.

Figure S2. The TIM barrel fold is a regular $(\beta-\alpha)_8$ repeat. The active site is located at the C-terminal ends of the 8 parallel β-sheets.

Figure S3. The NAD/NADP oxidoreductase Rossmann fold appears to be a $(\beta-\alpha)_8$ twisted sheet. A model for generating a Rossmann fold from a TIM barrel is proposed. As in the TIM barrel, the Rossmann fold active site is located at the C-terminal ends of the β-sheets.

Figure S4. The TOPRIM domain is a ~$(\beta-\alpha)_{4-5}$ repeat. The active site Mg is bound at the C-terminal ends of the β-sheets.

Figure S5. ABC ATPase transporters are Rossmann-like folds and ancient α/β proteins. The active site is located at the C-terminal ends of the β-sheets that form the Rossmann-like fold.

Figure S6. A kinase with a Rossmann-like fold $(\beta-\alpha)_5$. The active site is at the C-terminal ends of the β-sheets.

Figure S7. A β–α–β–α–β–α–β–(α)–β–α–β domain from a Swi-Snf ATPase HepA/RapA.[5]

Figure S8. A second β–α–β–α–β–α–β–α–β–α–β domain from a Swi-Snf ATPase HepA/RapA.[5]

Figure S9. Bacterial RNase HIII includes a TBP fold.[6,7]

Figure S10. A compact story of the genesis of life on earth focusing on 2-DPBB type RNAPs, RNAP GTFs, RNAP promoters and the RNAP II CTD. α/β fold proteins, required for metabolism and energy transduction, originated in the RNA-protein world and are dispersed to all cellular life (since LUCA; not shown). The red arrow indicates competition between Lokiarchaeota archaea and eukaryotes mostly won by eukaryotes.

References:

1. Nasir, A., Kim, K.M. & Caetano-Anolles, G. Lokiarchaeota: eukaryote-like missing links from microbial dark matter? *Trends Microbiol* (2015).
2. Spang, A. *et al.* Complex archaea that bridge the gap between prokaryotes and eukaryotes. *Nature* **521**, 173-9 (2015).
3. Koonin, E.V. Origin of eukaryotes from within archaea, archaeal eukaryome and bursts of gene gain: eukaryogenesis just made easier? *Philos Trans R Soc Lond B Biol Sci* **370**(2015).
4. Koonin, E.V. The origins of cellular life. *Antonie Van Leeuwenhoek* **106**, 27-41 (2014).
5. Liu, B., Zuo, Y. & Steitz, T.A. Structural basis for transcription reactivation by RapA. *Proc Natl Acad Sci U S A* **112**, 2006-10 (2015).
6. Brindefalk, B. *et al.* Evolutionary history of the TBP-domain superfamily. *Nucleic Acids Res* **41**, 2832-45 (2013).
7. Chon, H., Matsumura, H., Koga, Y., Takano, K. & Kanaya, S. Crystal structure and structure-based mutational analyses of RNase HIII from Bacillus stearothermophilus: a new type 2 RNase H with TBP-like substrate-binding domain at the N terminus. *J Mol Biol* **356**, 165-78 (2006).

Phagocytosis/endocytosis

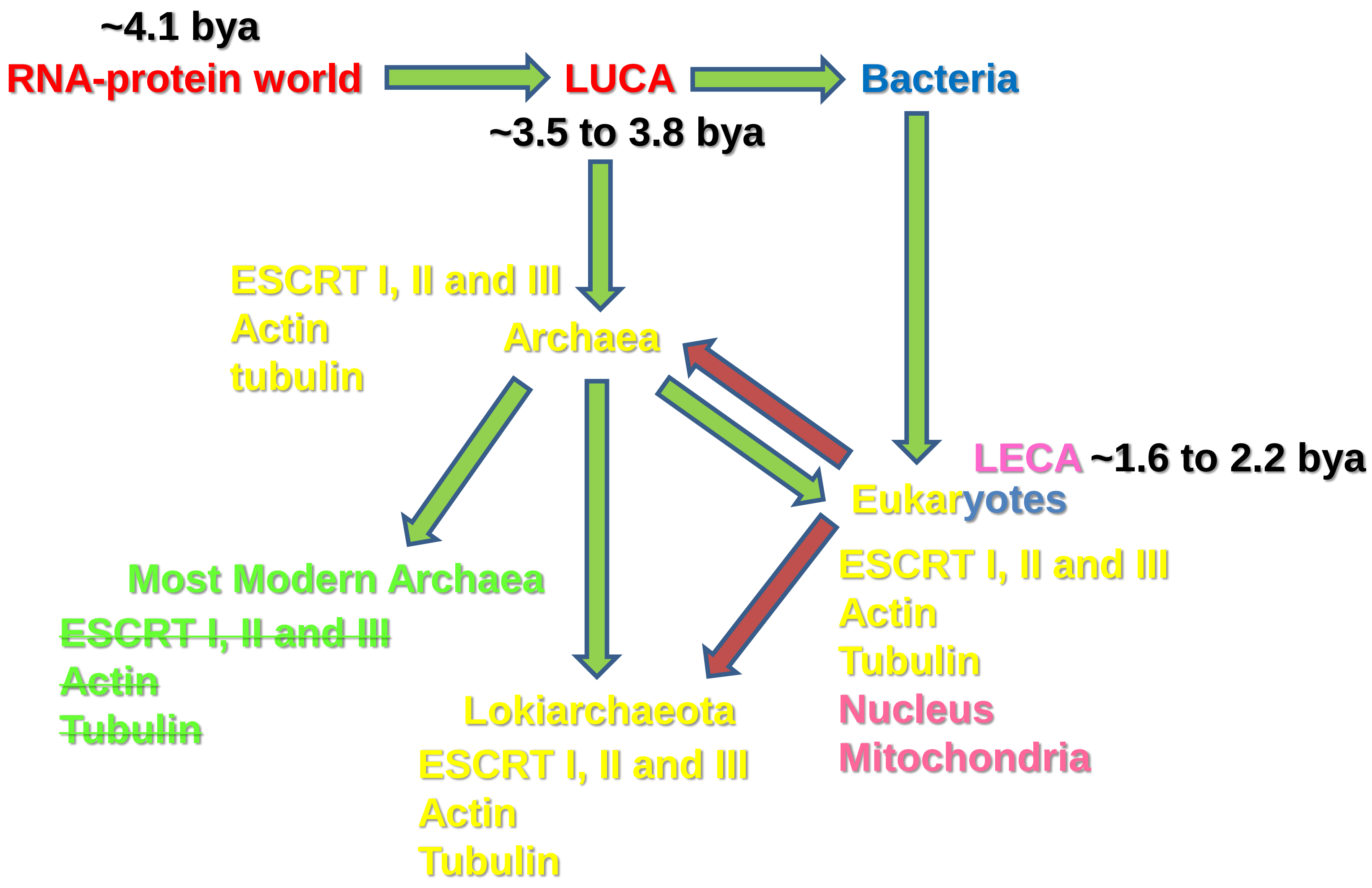

Figure S1.

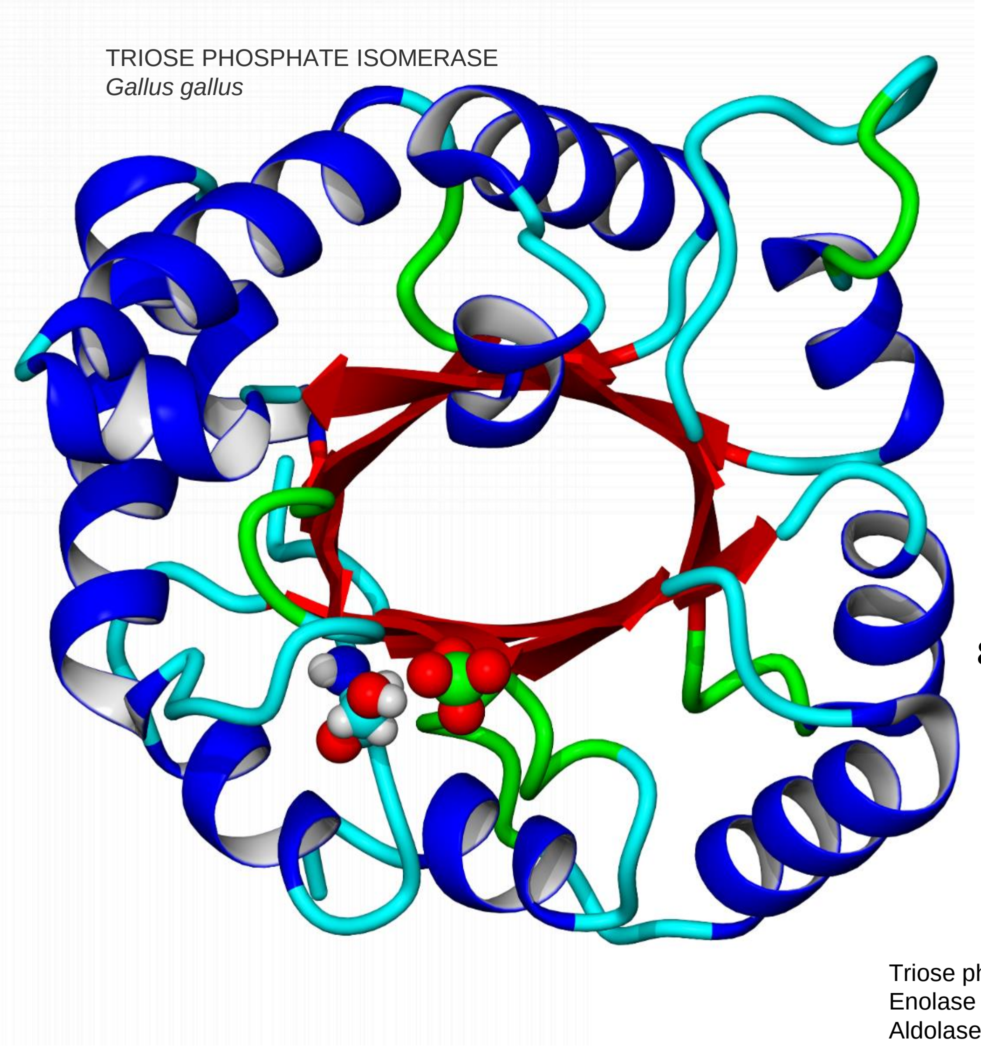

8TIM

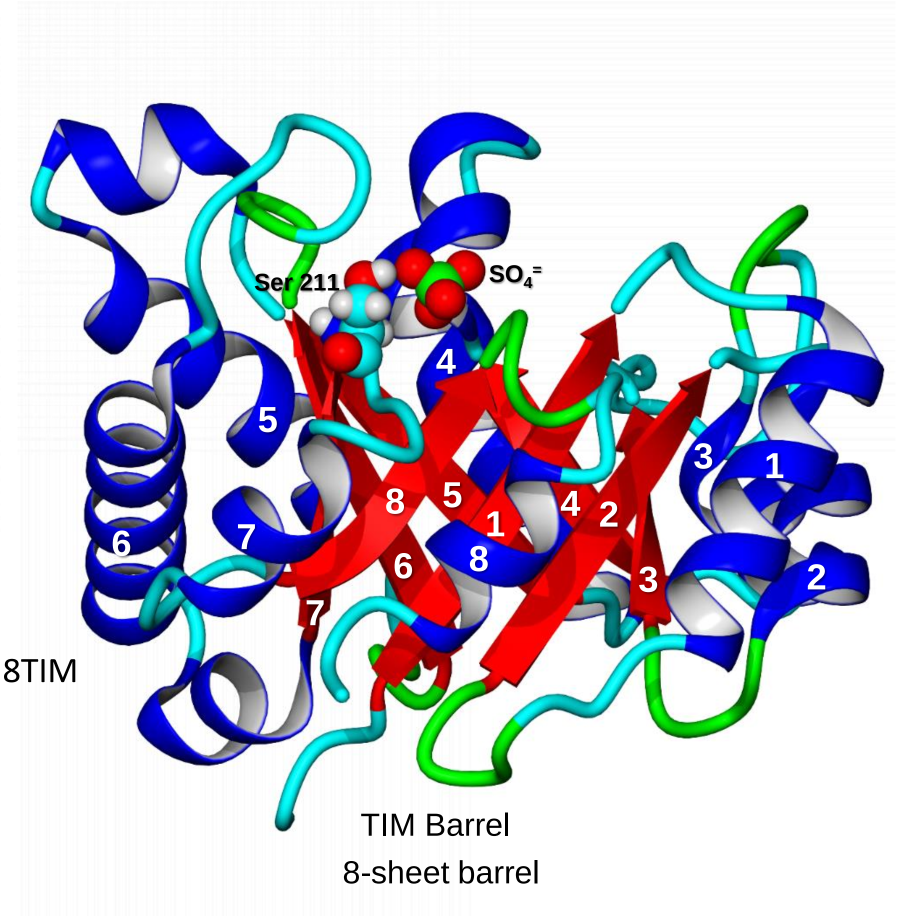

Triose phosphate isomerase
Enolase
Aldolase
Pyruvate kinase
FMN oxidoreductases
RuBisCo

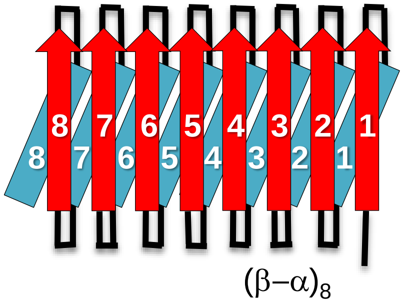

Figure S2.

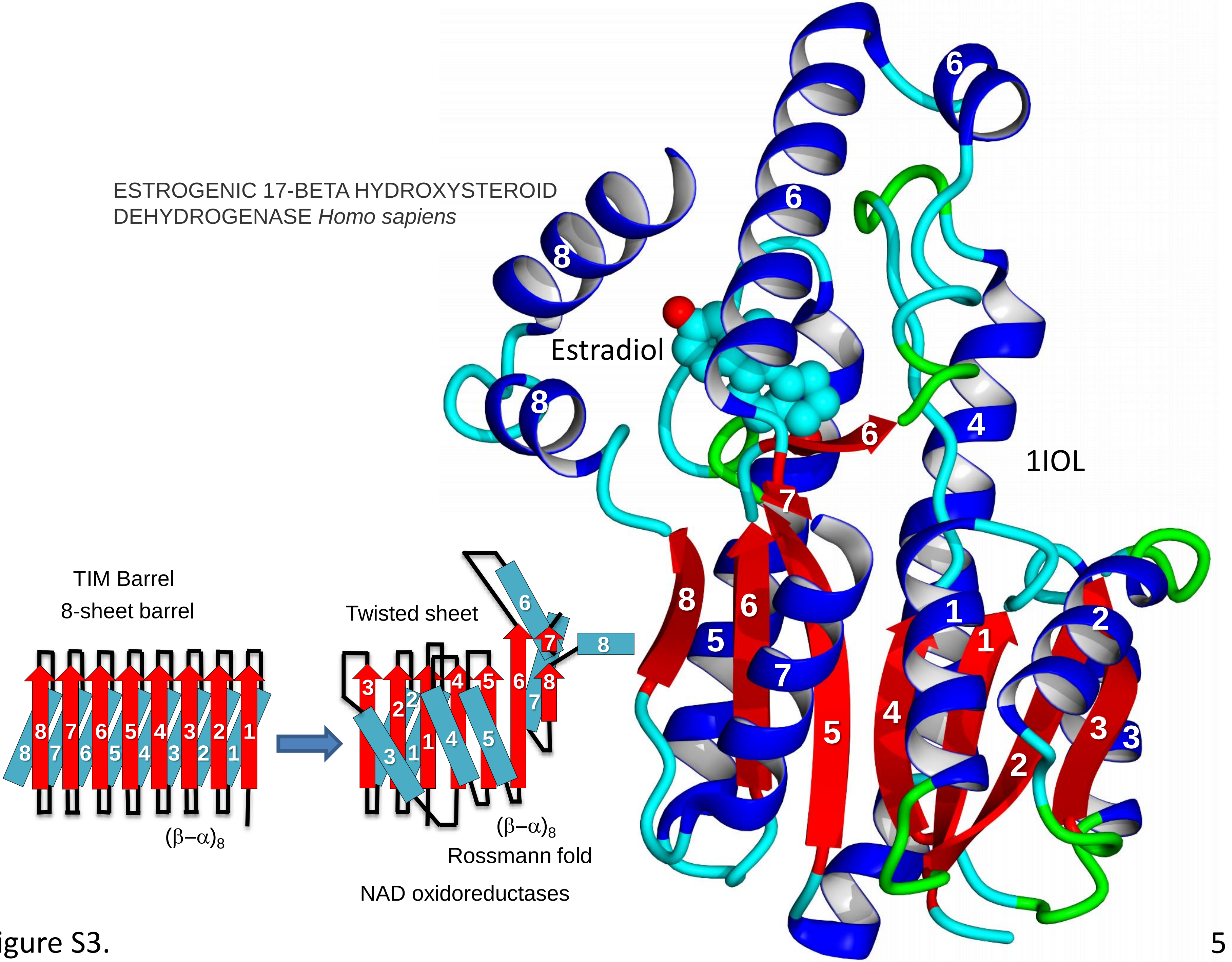
ESTROGENIC 17-BETA HYDROXYSTEROID
DEHYDROGENASE *Homo sapiens*
Estradiol
1IOL
TIM Barrel
8-sheet barrel
(β–α)8
Twisted sheet
(β–α)8
Rossmann fold
NAD oxidoreductases

Figure S3.

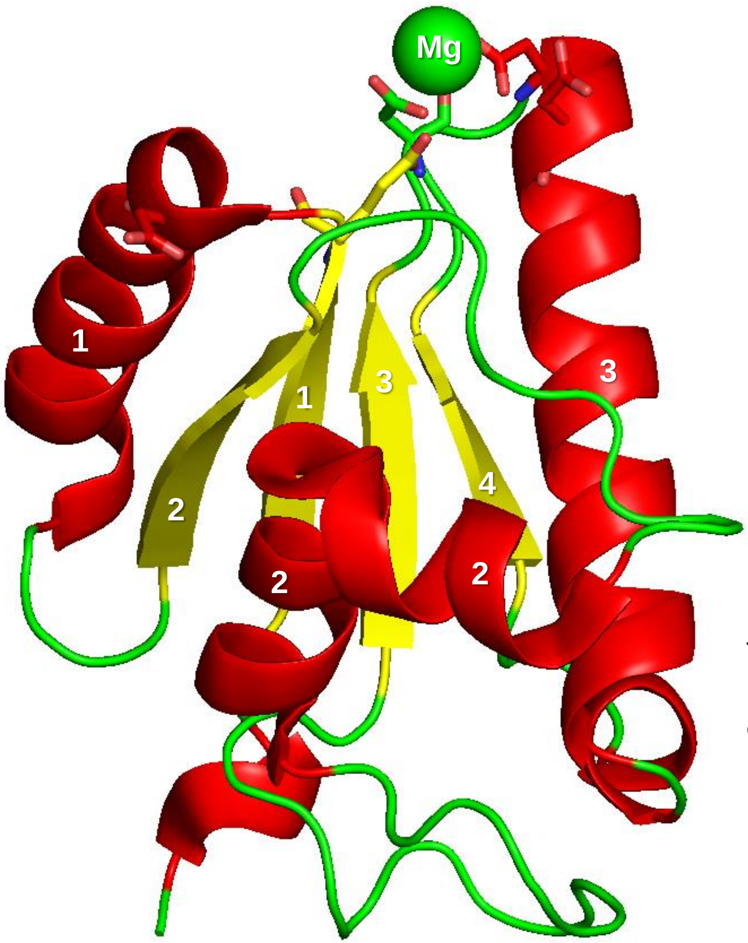

PDB 2RGR

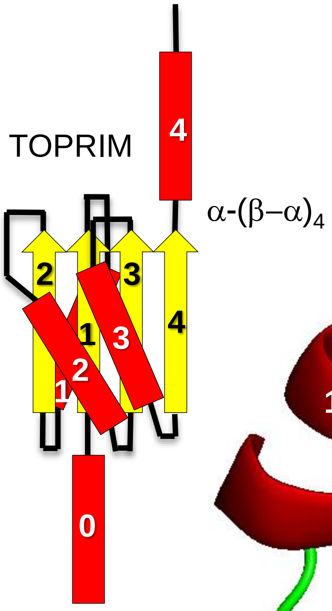

Topoisomerases
DNA primases
OLD nucleases
RecR/M DNA repair proteins

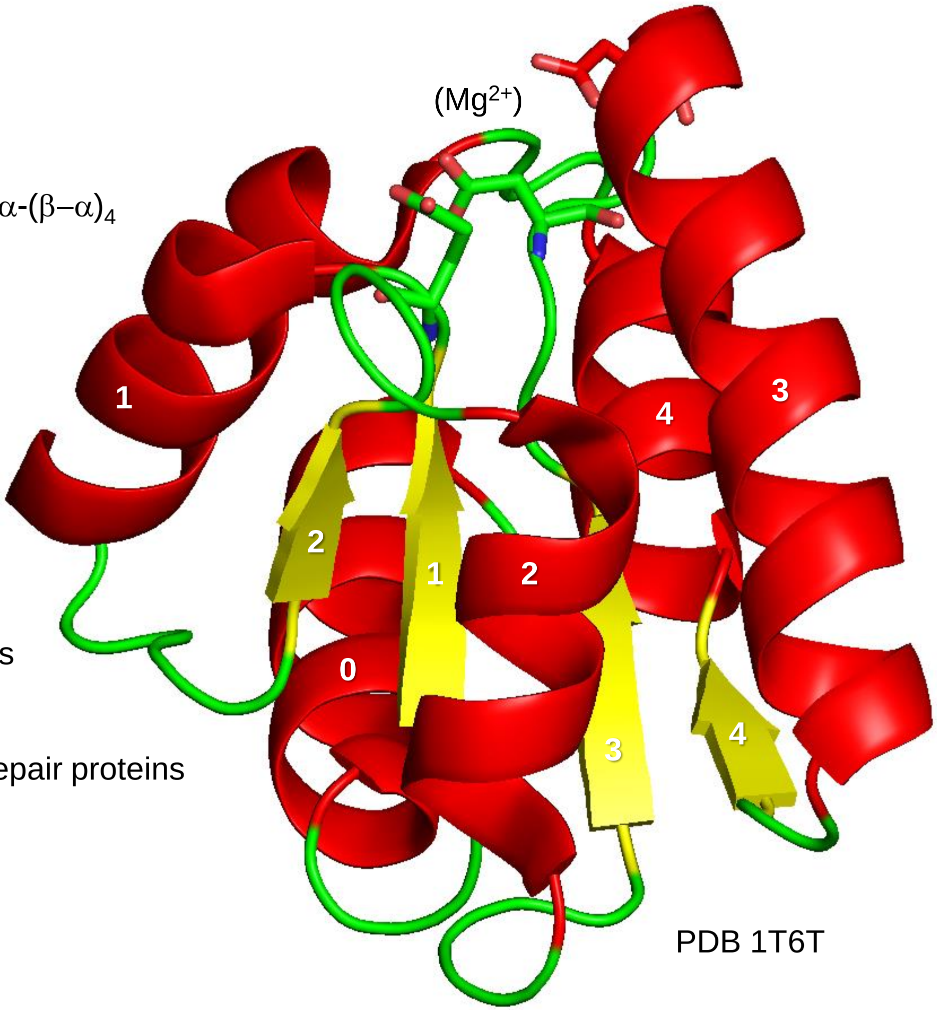

PDB 1T6T

Saccharomyces cerevisiae (yeast)
Type IIA DNA topoisomerase (gyrase type)

Aquaflex aeolicus (bacterial)
Putative protein

Figure S4.

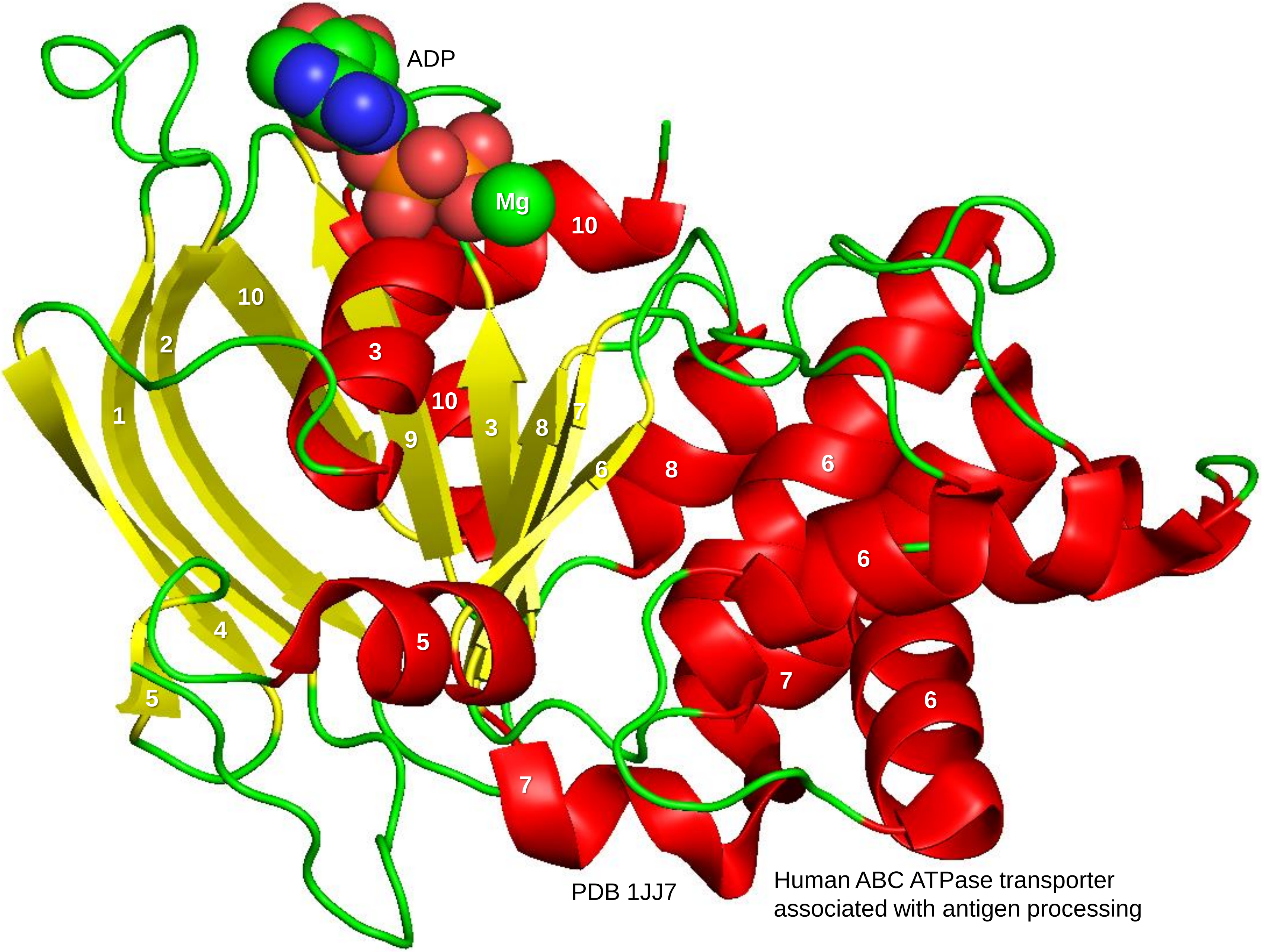
ADP
Mg
10
10
2
1
3
10
9
3
8
7
6
8
6
6
4
5
5
7
6
7
PDB 1JJ7
Human ABC ATPase transporter
associated with antigen processing

Figure S5.

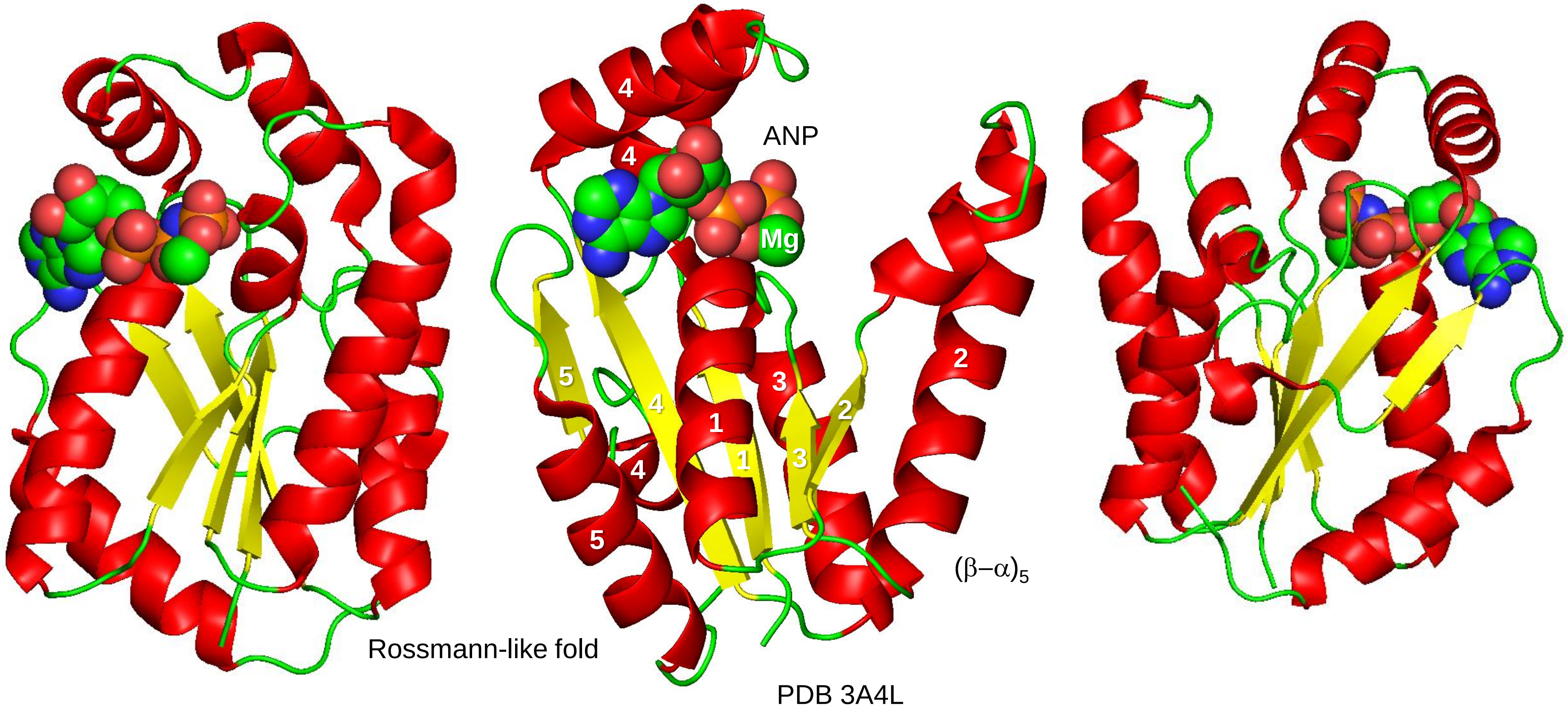

Methanocaldococcus jannaschii o-phosphoseryl-tRNA kinase

Figure S6.

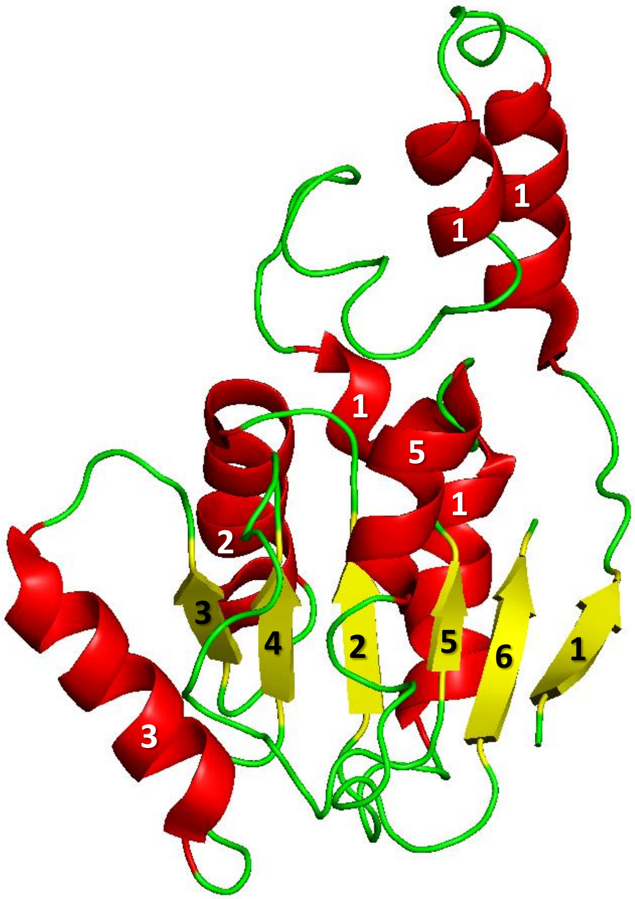

Escherichia coli HepA/RapA Swi-Snf ATPase $(\beta\!-\!\alpha)_6$ domain
PDB 4S20

Figure S7.

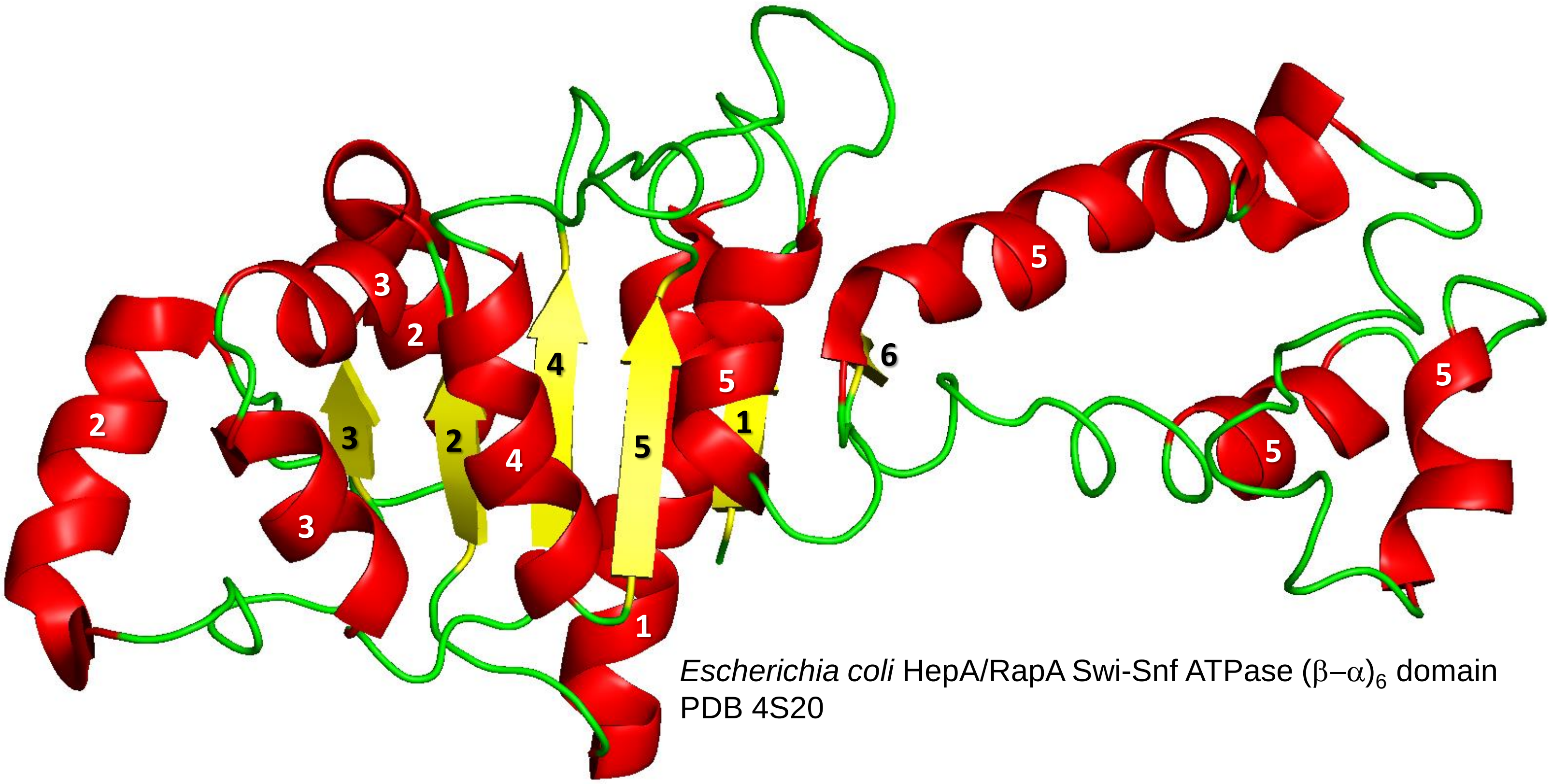

Escherichia coli HepA/RapA Swi-Snf ATPase $(\beta-\alpha)_6$ domain
PDB 4S20

Figure S8.

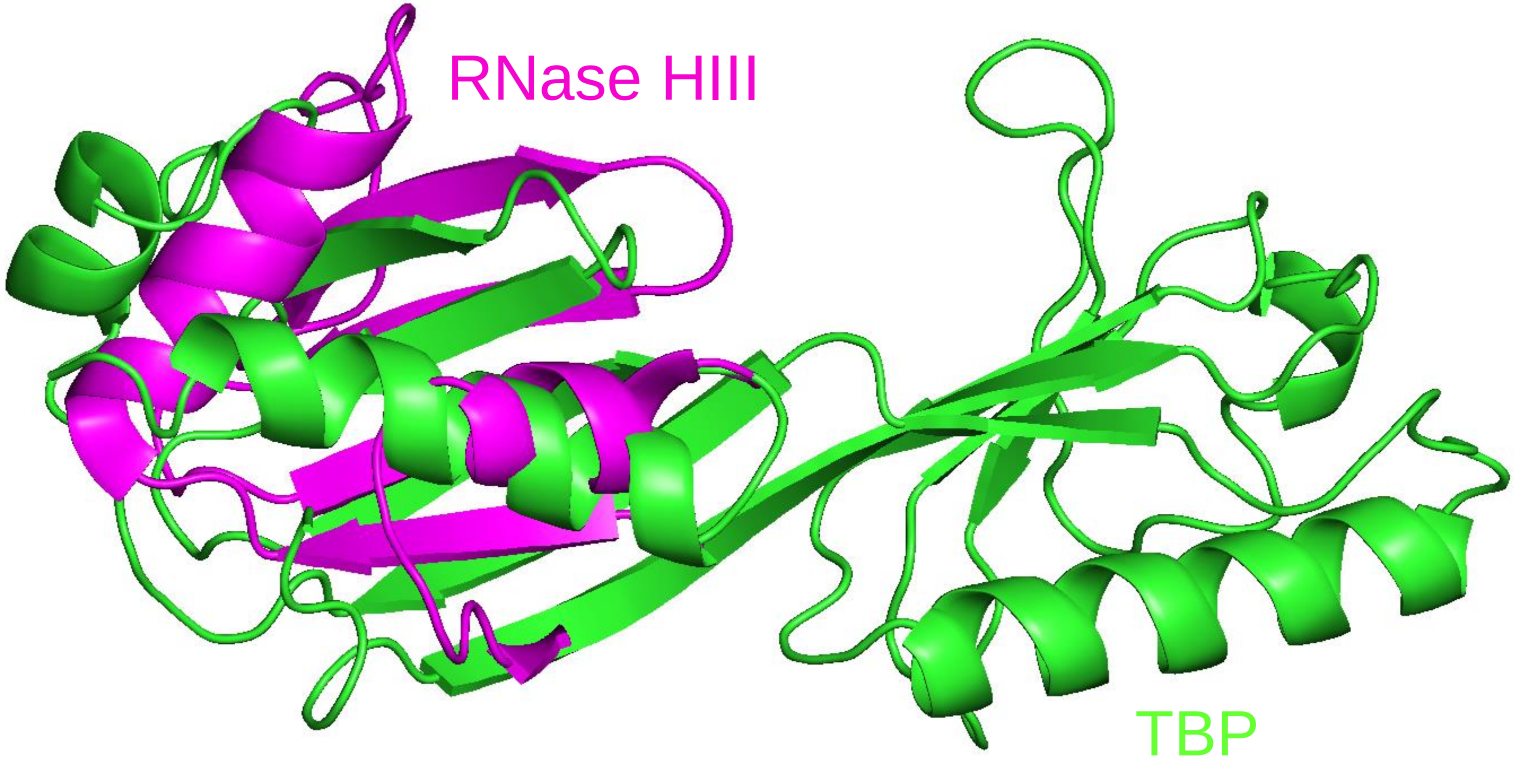

Figure S9.

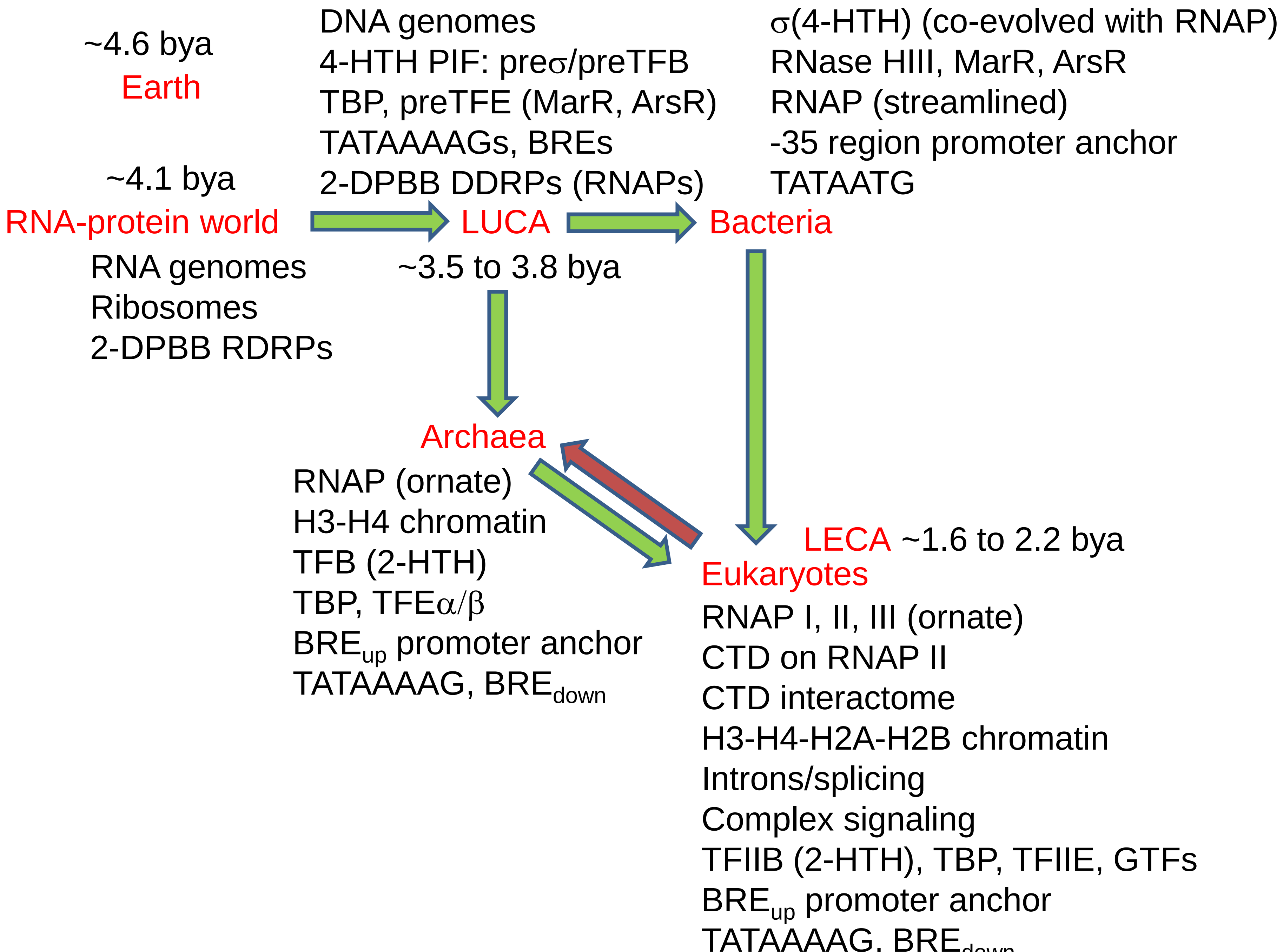
~4.6 bya
Earth
DNA genomes
4-HTH PIF: preσ/preTFB
TBP, preTFE (MarR, ArsR)
TATAAAAGs, BREs
2-DPBB DDRPs (RNAPs)
σ(4-HTH) (co-evolved with RNAP)
RNase HIII, MarR, ArsR
RNAP (streamlined)
-35 region promoter anchor
TATAATG
~4.1 bya
RNA-protein world
LUCA
Bacteria
RNA genomes
Ribosomes
2-DPBB RDRPs
~3.5 to 3.8 bya
Archaea
RNAP (ornate)
H3-H4 chromatin
TFB (2-HTH)
TBP, TFEα/β
BRE_{up} promoter anchor
TATAAAG, BRE_{down}
LECA ~1.6 to 2.2 bya
Eukaryotes
RNAP I, II, III (ornate)
CTD on RNAP II
CTD interactome
H3-H4-H2A-H2B chromatin
Introns/splicing
Complex signaling
TFIIB (2-HTH), TBP, TFIIE, GTFs
BRE_{up} promoter anchor
TATAAAG, BRE_{down}

Figure S10.
